# The prognosis of patients with dissociated virological and immunological responses to HAART

**DOI:** 10.1186/1758-2652-13-S4-P55

**Published:** 2010-11-08

**Authors:** DJ Jevtovic, DR Salemovic, JT Ranin

**Affiliations:** 1 Infectious Diseases University Hospital, Belgrade, Serbia and Montenegro

## Background

While HAART allows for the reconstitution of immune functions in most treated HIV patients, failure to achieve a significant increase in circulating CD4+ T cells despite undetectable viremia occurs.

## Methods

A retrospective study was conducted to evaluate the treatment outcome in a subgroup of 232 patients who after 3.1 years of treatment had not achieved desirable immune reconstitution despite a good virological response to HAART.

## Results

After a further 3.5±2.7 years of HAART, 41 (17.7%) patients achieved immune reconstitution (681.4±172.7 CD4 cells/µL), while 191 (82.3%) patients did not (306.6±109.16 cells/µL); the difference in the achieved CD4 counts between these subgroups was significant (P<0.01). One patient experienced treatment failure. Eleven patients died to the end of follow-up, of which ten with a continuously dissociated response. Factors associated with immune recovery included, usage of PIs and of drugs from all three classes (OR 2.1, 95% CI 1.0-4.2, P=0.037 and OR 5.1, 95% CI 1.4-18.7, P=0.013, respectively), and a rise in CD4 count to over 200 cell/µL after the first 3.1 years of treatment (OR 2.8, 95% CI 1.1-6.6, P=0.019). Achievement of a rise in CD4 count to over 200 cell/µL after the first 3.1 years of treatment, and usage of all three drug classes were independent predictor of immune reconstitution in the following period.

## Conclusions

If patients on HAART reach CD4 cell counts of above 200 cells/µL in the first three years, immune recovery is possible after at least six years of treatment, particularly if treated with drugs from all three classes.

